# Persistent fecal occult blood due to the small intestinal metastasis of pleomorphic lung carcinoma

**DOI:** 10.1093/jscr/rjac043

**Published:** 2022-02-19

**Authors:** Takaya Suzuki, Masafumi Noda, Akihiro Yamamura, Hisashi Ohishi, Hirotsugu Notsuda, Shunsuke Eba, Ryota Tanaka, Naoki Tanaka, Takashi Kamei, Michiaki Unno, Yoshinori Okada

**Affiliations:** 1 Department of Thoracic Surgery, Institute of Development Aging and Cancer, Tohoku University, Sendai 980-0872, Japan; 2 Department of Surgery, Tohoku University Graduate School of Medicine, Sendai 980-0872, Japan

## Abstract

The gastrointestinal tract is one of the locations that lung cancers cause metastasis. A 70-year-old male underwent right lower lobectomy while presenting fecal occult blood with a preoperative colonoscopy showing colon polyps as the cause. The pathological diagnosis was pleomorphic carcinoma of the lung, with stage pT3N0M0. Seven months after the lung surgery, the patient presented with sudden-onset abdominal pain and severe anemia. Computed tomography scanning revealed a large mass in the abdominal cavity, and subsequent intestinal endoscopy demonstrated jejunum tumors. Partial jejunum resection was successfully performed. The patient developed multiple peritoneal nodules suggesting metastatic tumors but well responded to an immune checkpoint inhibitor. It can be challenging to diagnose gastrointestinal metastasis in routine radiography; therefore, endoscopic examination, including the small intestine, might be an important option when a lung cancer patient with advanced clinical stage presents with abdominal symptoms, including fecal occult blood.

## INTRODUCTION

Symptomatic gastrointestinal (GI) metastasis of non-small cell lung cancer is rare, accounting for 0.17 [1], 0.26 [2], 1.77% [3] or roughly under 2% of non-small cell lung cancer cases. The small intestine is the most common GI metastatic site for lung cancer [4]. The typical manifestation includes abdominal pain; however, patients with intestinal tumors often lack specific symptoms, which could result in delayed diagnosis. It is challenging to demonstrate metastatic intestinal tumors preoperatively, as routine GI tract endoscopy does not inspect the small intestine. Here, we report a case of surgically resected pleomorphic carcinoma of the lung presenting persistent fecal occult blood, who subsequently showed multiple GI tract.

## CASE REPORT

**Figure 1:**
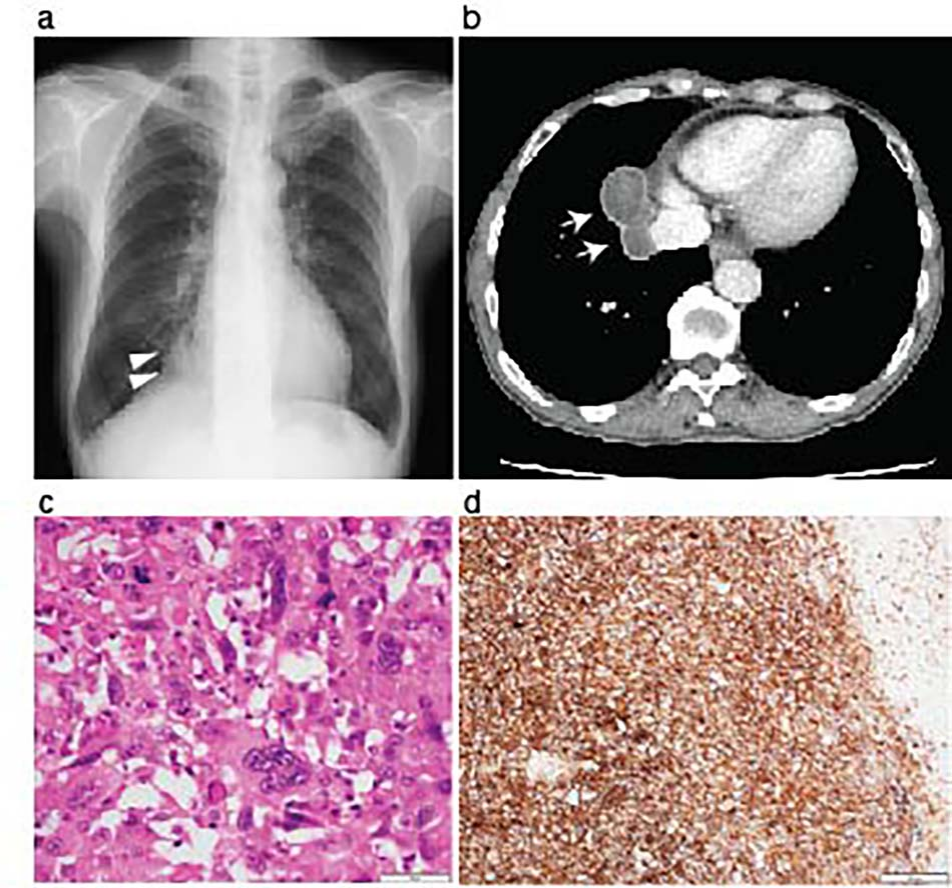
Resection of the lung pleomorphic carcinoma. A chest X-ray on admission showed a nodule in the right lung field (arrowheads) (**a**). Enhanced CT-scanning showed a well-circumvented nodule along the inferior vena cava (**b**, arrows). Hemotoxylin and eosin staining of the resected tumor showed the mixture of spindle and giant malignant cells consistent with the pleomorphic carcinoma (**c**). Immunohistochemistry revealed high PD-L1 expression with a tumor proportion score > 50%. Scale bar, 50 μm (c), and 200 μm (**d**).

A 70-year-old man was referred to us for investigating an abnormal shadow in the right lower lung lobe in a computed tomography (CT) scanning. He had diabetes mellitus and an old myocardial infarction. The laboratory test of the patient showed slight anemia (Hemoglobin 11 mg/dL and red blood cell count 3.44×10^6^/μL) without any coagulopathy. The patient also showed fecal occult blood. The preoperative colonoscopic examination revealed adenomatous polyps and a diverticulum in the descending colon that were considered the cause of fecal occult blood at that time. Tumor markers were as follows; Carcinoembryonic antigen 4.3 ng/ml, Neuron-specific enolase 7.2 ng/ml, Squamous cell carcinoma antigen 1.0 ng/ml and Pro-gastrin-releasing peptide 16.4 ng/ml. The chest X-ray showed a nodule in the right lower lung area (Fig. 1a). The chest computed tomography revealed a tumor in the right lower lobe, which was well-circumvented, 5.9 centimeters in diameter, and adjacent to the diaphragm and the inferior vena cava (Fig. 1b). The patient underwent a curative right lower lobectomy and mediastinal lymph node dissection with combined resection of the pericardium and diaphragm. The defect was reconstructed by suturing Gore-Tex™ sheet. The histological diagnosis of the tumor was pleomorphic carcinoma. The pathological stage was pT3N0M0, pStage IIB, as the tumor involved the pericardium. The patient was discharged on postoperative Day 10. Programmed death-ligand 1 (PD-L1) expression was >50% with immunohistochemistry (Fig. 1d). Being the pathological stage IIB, adjuvant chemotherapy with cisplatin and vinorelbine was indicated. The first dose was administered a month after the primary operation; however, the patient showed grade 4 leucopenia and nausea. The adjuvant chemotherapy was therefore discontinued.

**Figure 2:**
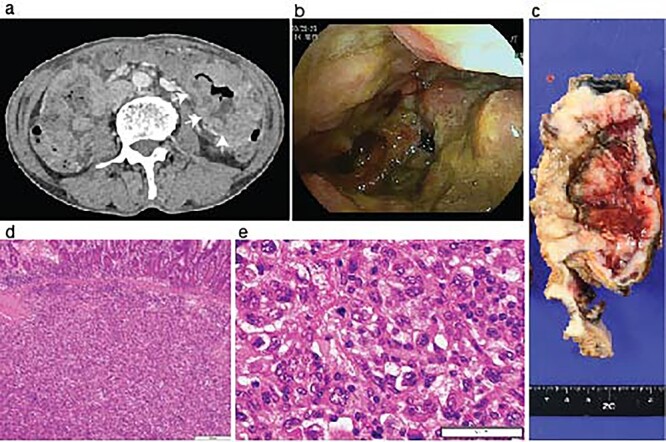
Identification of a metastatic intestinal tumor after lung surgery. An abdominal enhanced CT-scanning revealed a large mass lesion suggestive of small bowel involvement (**a**, arrows). A jejunum tumor was found in intestinal endoscopy (**b**). A surgical specimen of the metastatic lesion of the jejunum (**c**). H&E staining of the jejunum tumor showed submucosal malignant cells (**d**) with pleomorphic features compatible with the primary pleomorphic lung carcinoma (**e**).

During the follow-up period, the patient presented with progressive anemia and general fatigue. Seven months after the lung surgery, the blood hemoglobin dropped to 6.8 mg/dl from 10.6 mg/dl at 2 months after the lung surgery. The patient was hospitalized, and a red blood cell infusion was performed. As fecal occult blood was consistently observed after the lung operation, another endoscopic examination of the upper and lower digestive tract was scheduled. In the meantime, the patient demonstrated a sudden onset of left side severe abdominal pain. The emergent enhanced CT scanning revealed a large abdominal mass in the left abdominal cavity (Fig. 2a). The subsequently performed small intestinal endoscopy revealed a tumor in the jejunum (Fig. 2b), and the biopsy of the tumor demonstrated the metastasis of the lung cancer. The partial resection of the jejunum including the metastatic lesion followed by end-to-end anastomosis was performed 8 months after the lung operation. The pathological diagnosis of the jejunum tumor was pleomorphic carcinoma, which was identical to the primary lung cancer (Fig. 2c-e). After 1 month of the second surgery, the patient again showed abdominal pain. The gastroscopy examination revealed a gastric tumor in the stomach wall with blood wheezing. Distal gastrectomy was performed 10 months after the lung operation. Some peritoneal nodules suspected of dissemination were also resected. The pathological diagnosis of the gastric tumor and peritoneal nodules was also pleomorphic carcinoma.

**Figure 3:**
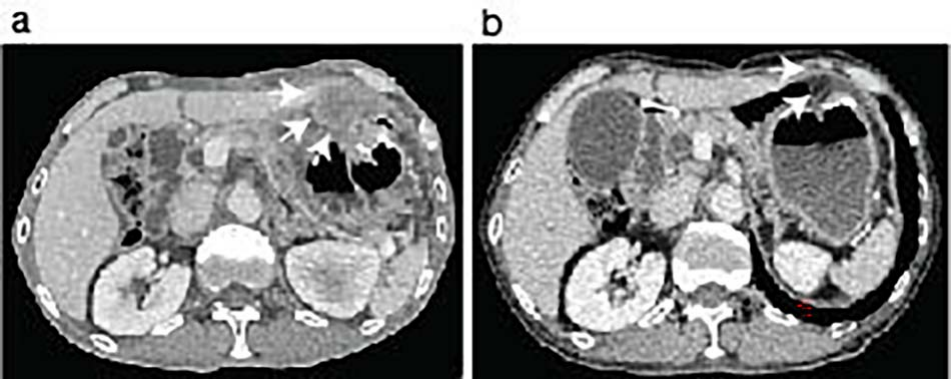
ICI therapy after the abdominal surgery. The patient showed peritoneal nodules (arrows) in the first follow-up CT scan after the last abdominal surgery (**a**). The peritoneal nodules were almost disappeared after 6 months following Pembrolizumab treatment (**b**, arrows).

The patient was discharged 3 weeks after the second abdominal surgery. Because the growth of the peritoneal dissemination of the metastatic pleomorphic carcinoma was observed in the follow-up CT scans (Fig. 3a) at 2 months of the second abdominal surgery, the patient underwent immune checkpoint inhibitor (ICI) therapy using pembrolizumab after the final abdominal surgery. The ICI was significantly effective (Fig. 3b), and the patient has remained cancer-free state at 26 months since the first surgery.

## DISCUSSION

Pleomorphic carcinoma of the lung is a relatively rare pathologic diagnosis that accounts for approximately 0.1–0.4% of non-small cell lung cancer [5]. The prognosis of pleomorphic carcinoma has been reported to be worse than adenocarcinoma or squamous cell carcinoma [6] because of the aggressive tumor progression, including distant organ metastasis [7]. Any type of non-small cell lung carcinoma can metastasize to distant organs, including the GI tract; however, a study has shown that pleomorphic carcinomas are significantly associated with GI tract metastasis than other types of non-small cell lung carcinomas [8]. In the present case, abdominal symptoms were not apparent in the first peri-operative period. It was just fortunate that the patient did not present GI tract perforation or ileus, which could have occurred during the follow-up period. Considering the difficulties in diagnosing GI tract metastasis with routine follow-up imaging, including a CT scan, it seems essential to include an endoscopic screening of the GI tract when there are findings suggestive of GI tract involvement such as fecal occult blood.

Interestingly, previous observational studies demonstrated that the non small cell lung carcinoma, including pleomorphic carcinoma that caused intestinal metastasis presented prominent PD-L1 expression [9, 10]. The PD-L1 expression of the lung tumor in this case was also high (Fig. 1d), and the patient responded to Pembrolizumab for the treatment of metastatic peritoneal lesions. The surgeries for the primary lung tumor and subsequent abdominal metastatic lesions were necessary to achieve the local tumor management and might have helped the patient to be able to undergo ICI treatment in good performance status.

## CONCLUSION

Sustained fecal occult blood can be the manifestation of GI metastasis in lung cancer patients. It can be challenging to diagnose GI metastasis in routine radiography; therefore, endoscopic examination of the entire digestive tract, including the small intestine, might be an important option when an advanced-stage lung cancer patient presents with abdominal symptoms including fecal occult blood.
